# Supplementary material for: Post-drought decline of the Amazon carbon sink
Source: Nat Commun. 2018 Aug 9;9:3172. doi: 10.1038/s41467-018-05668-6 (PMC6085357; doi:10.1038/s41467-018-05668-6)
Supplement: Supplementary file 1 — Supplementary Information [file 41467_2018_5668_MOESM1_ESM.pdf]

# Post-drought Decline of the Amazon Carbon Sink

Yang et al.

## Supplementary Discussion

The GLAS-derived tree height map in 2003 might have an overall underestimation of the dense forests of Amazon. A previous study[1] reported that the retrieval of 2003 has a range within 81 meters. If the GLAS instrument triggers the land retrieval earlier, there is a probability to truncate the return signal without capturing the entire waveform for tall vegetation and steep slopes. The GLAS team fixed the waveform truncation problem after 2004, as "the waveform compression scheme was implemented to increase the land height range to 150 m". Meanwhile, due to the relatively larger footprint size in 2003, top canopy heights may also be overestimated. These differences in data acquisition and quality map impact the estimated statistics of height and the carbon stocks towards a slight overestimation that can potentially reduce the carbon change between 2003 and 2004. Therefore, data in 2003 should be used with caution and the associated carbon stock change may be an underestimation.

On the other hand, GLAS data in 2008 stands out to be less accurate in regional coverage due to much fewer good-quality data (~30% of the average number of shots for other years) available over the entire Amazon with clustered observations in limited regions **(Supplementary Fig. 4)**. Therefore, significant biases could exist in the regional estimation when gap-filling is not applied. Due to these artifacts existing in the years 2003 and 2008, we excluded them in building carbon models (see **Methods**).

## Supplementary Figures and Tables

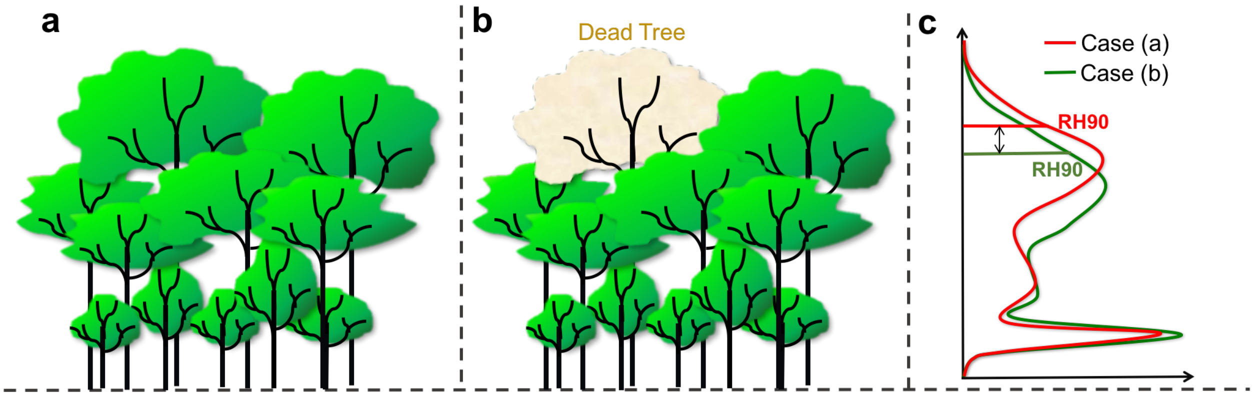


**Supplementary Figure 1.** Illustration of the RH90 metric change derived from GLAS waveforms. **(a)** Forest canopy structure without disturbance; **(b)** the same forest canopy as (a) except a dead tree with defoliated tree crown; and **(c)** comparison of LiDAR waveforms and the change of RH90 from case (a) to case (b).

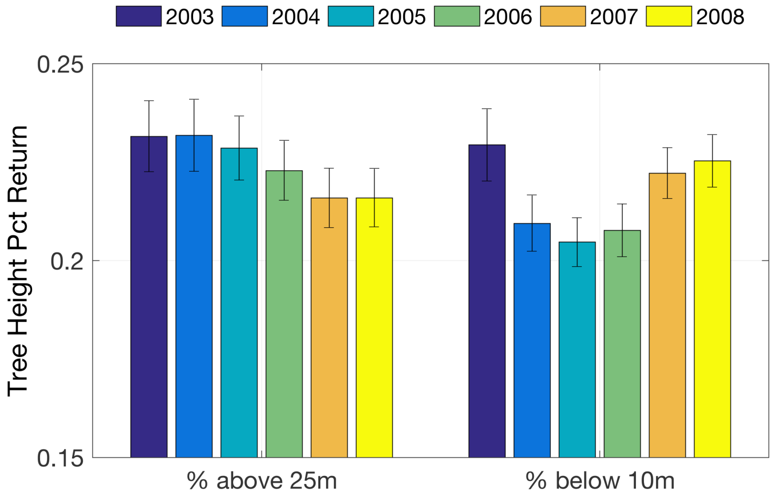


**Supplementary Figure 2**. Basin-wide inter-annual changes of the mean fraction of GLAS waveform return above or below a certain fixed height above ground. The choices of the fractions **above 25** and **below 10** meters were determined by the approximate mean values of RH90 and RH30, but more accurately, the numbers are closer to the metrics of RH80 and RH20. The error bars stand for 95% confidence intervals.

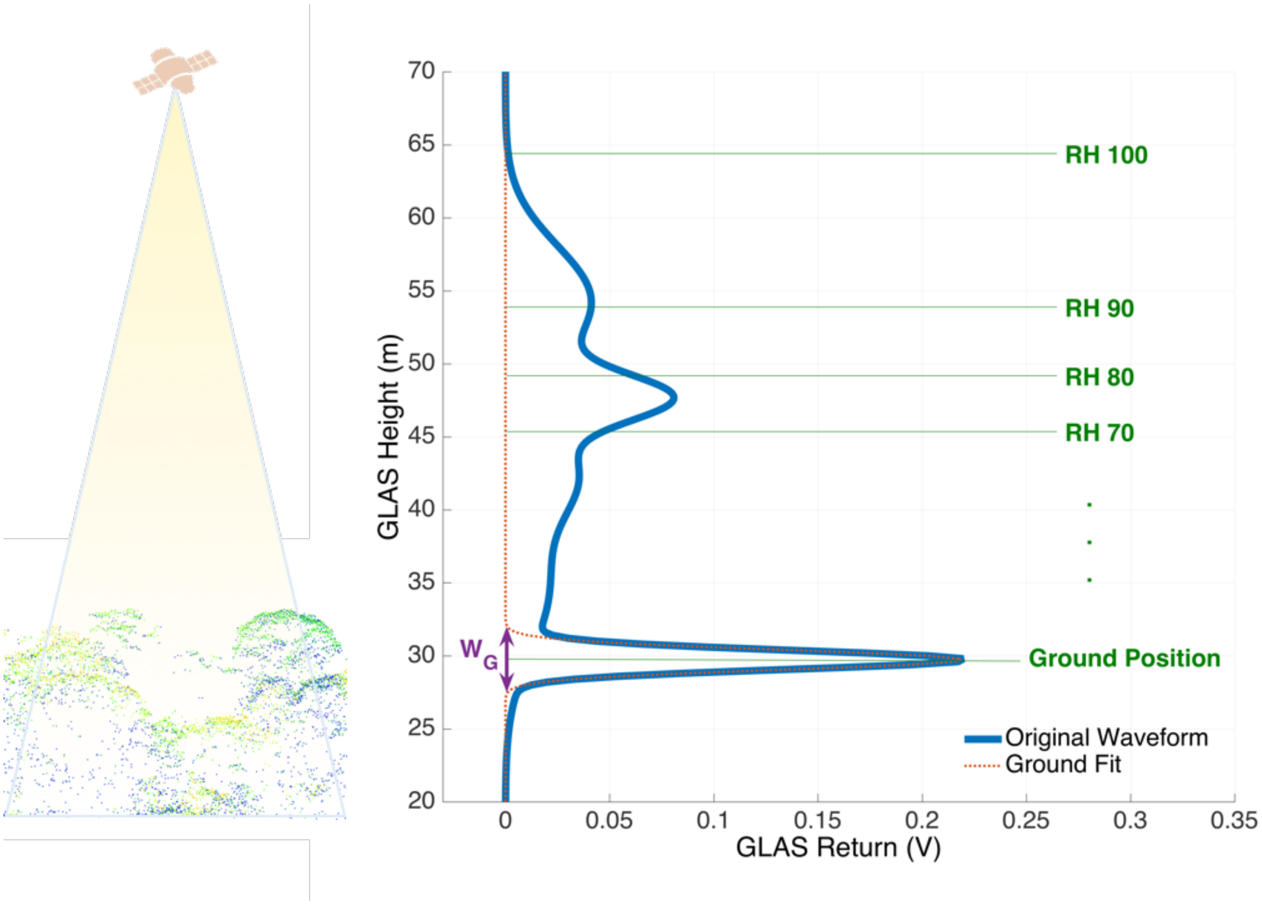


**Supplementary Figure 3.** Illustration of GLAS LiDAR waveform retrievals. The right panel is an example waveform reconstructed from GLAH14 product. RH metrics and $W_G$ were defined in Supplementary Text 1.2.1.

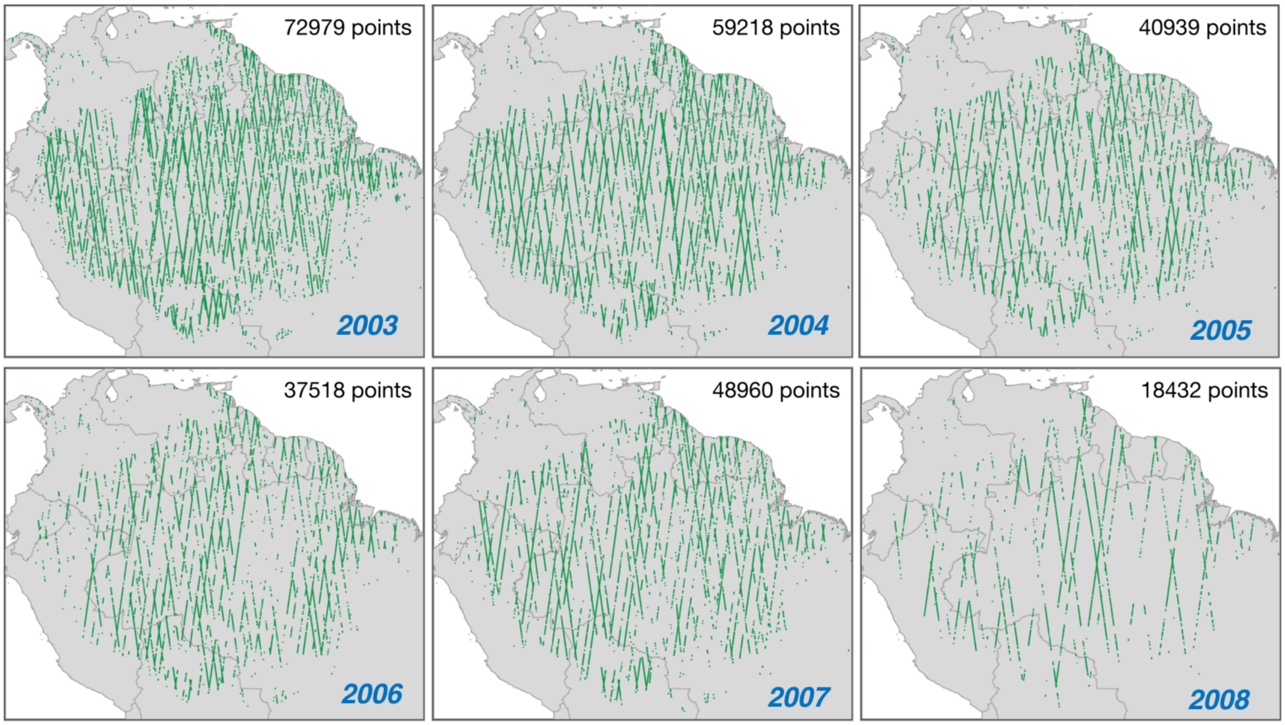


**Supplementary Figure 4.** Locations for all available GLAS observations after performing all GLAS filters in the Amazonia for each year. GLAS data shown in the figure were obtained from the GLAH14 product[2].

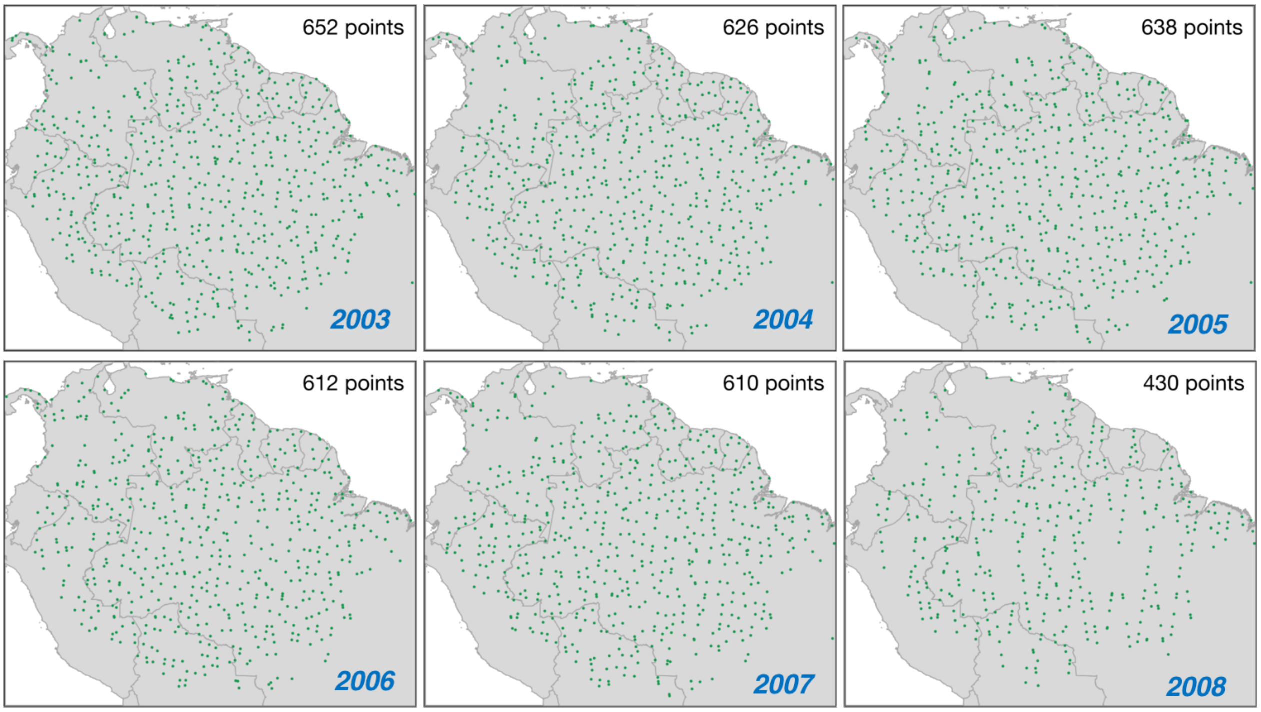


**Supplementary Figure 5.** One scenario of random sampling from valid GLAS observations without gap filling. GLAS data shown in the figure were obtained from the GLAH14 product[2].

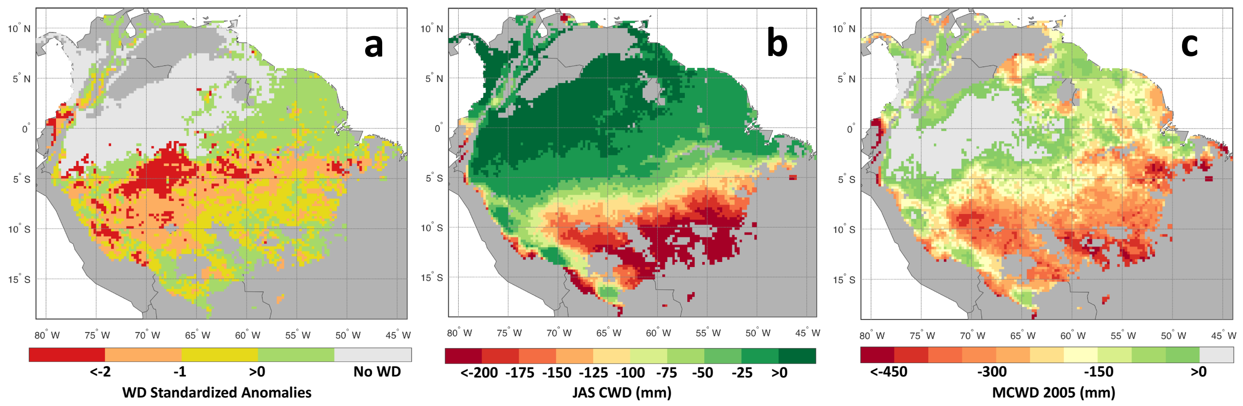


**Supplementary Figure 6.** Cumulative water deficit (CWD) maps derived from rainfall (TRMM) data[3]. **(a)** JAS (average data for July, August and September) CWD anomaly in 2005 with the JAS CWD from 2000 to 2009 as the base period; **(b)** Mean JAS CWD averaged over the base period (2000-2009); **(c)** Maximum monthly CWD in 2005.

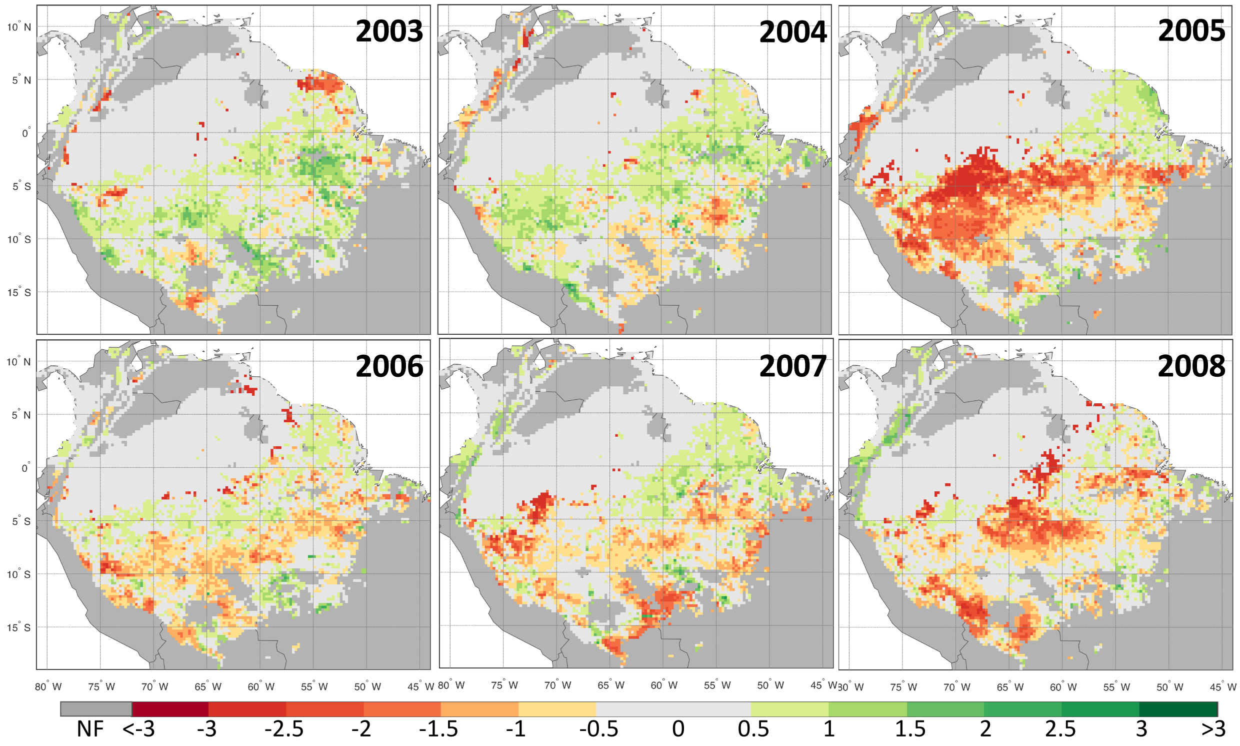


**Supplementary Figure 7.** The spatial distribution of JAS CWD anomalies from 2003 to 2008 using rainfall data[3]. The anomalies were calculated using mean and standard deviation over the years 2000-2009 as the base period.

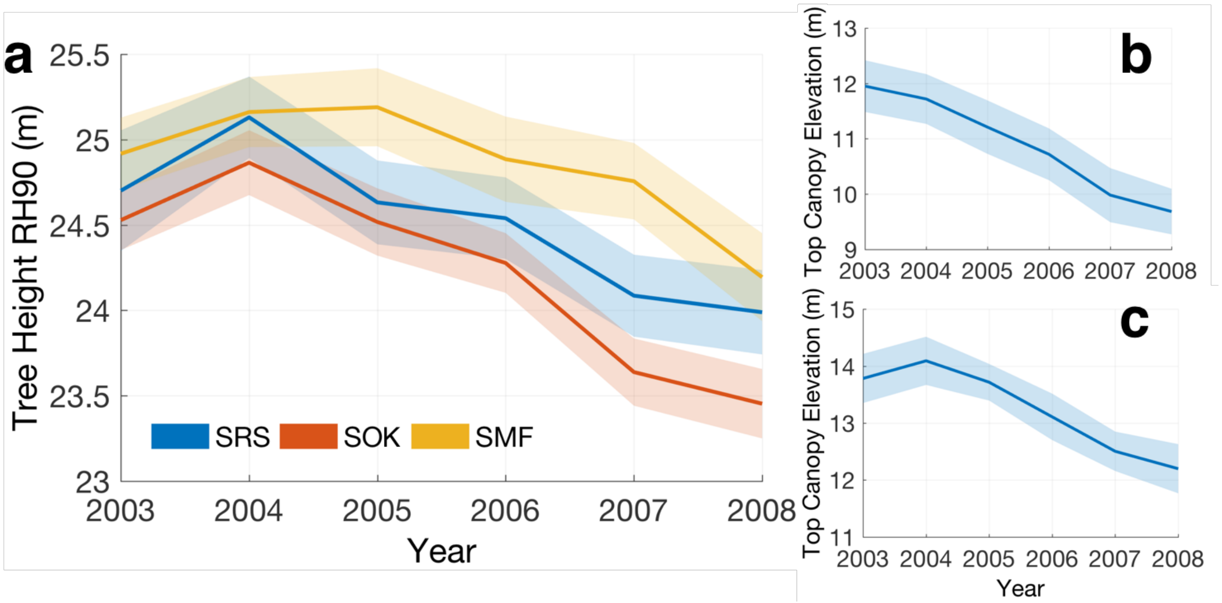


**Supplementary Figure 8.** Tests of GLAS-derived Height Metrics in Amazon. **(a)** RH90 inter-annual change of the entire amazon using 3 different methods, including the SRS, SOK and SMF methods (see **Supplementary Information**). And annual changes of GLAS-derived Top Canopy Height $H_G$ using **(b)** the SRS method and **(c)** the SMF method.

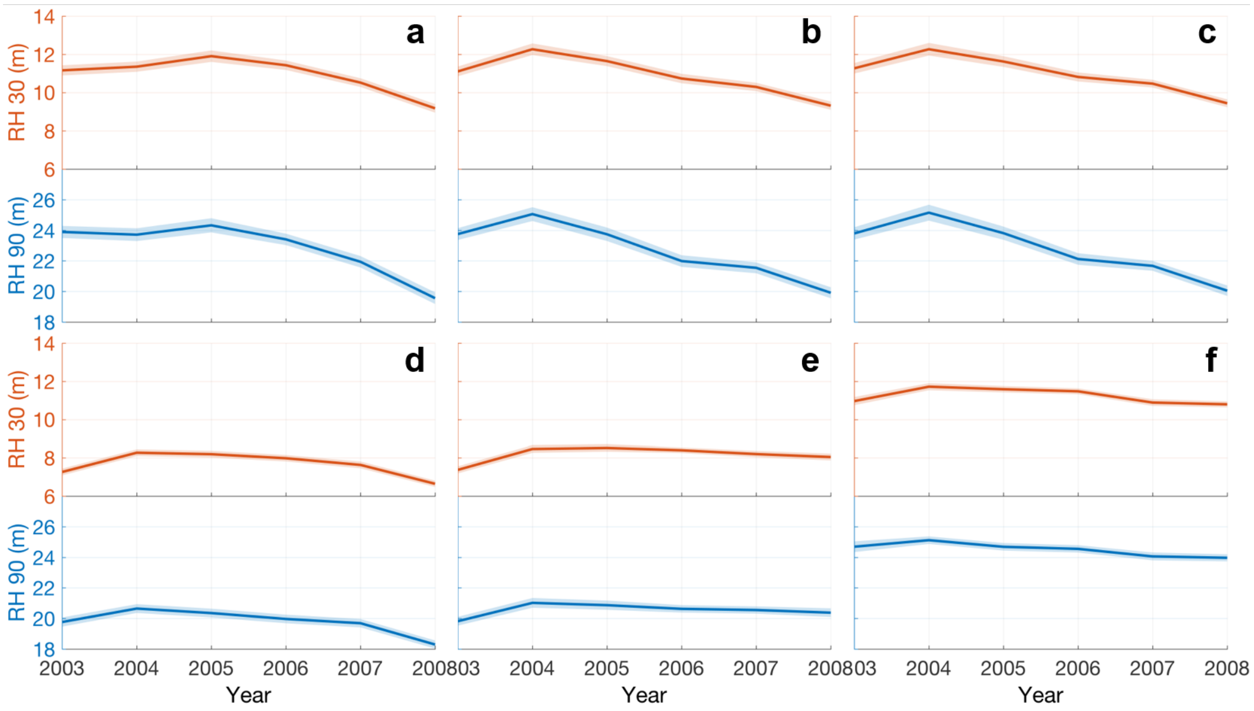


**Supplementary Figure 9**. Evolution of the GLAS data filtering, shown as the annual changes of tree height over Amazon after **(a)** the LC/VCF filter; **(b)** Seasonal Filter; **(c)** Saturation Filter; **(d)** 2-peak Filter; **(e)** Cloud filter; and **(f)** Slope filter. The Changes of GLAS RH metrics (RH 90 and 30) were shown as the average estimation using the SRS sampling method over Amazon forests.

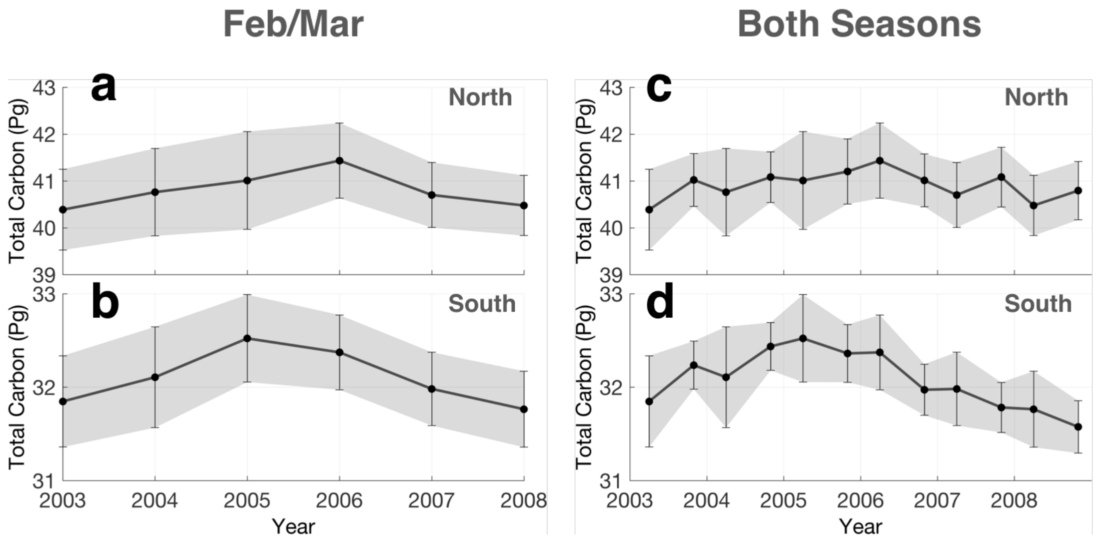


**Supplementary Figure 10**. Total Carbon changes derived from different GLAS seasons. **(a)** Inter-annual total carbon changes in North using GLAS data retrieved from the Feb/Mar season; **(b)** Inter-annual total carbon changes in South using GLAS data retrieved from the Feb/Mar season; (c) Combing panel (a) and Fig. 2b of the main manuscript; (d) Combing panel (b) and Fig. 2c of the main manuscript. Estimations in this figure were derived from the SRS method for faster processing. The error bars (shaded area) stand for 95% confidence intervals.

**Supplementary Table 1**: Interannual changes of GLAS top canopy structure (RH90) and carbon storage (average AGB and Total Carbon) in different regions of Amazon. The uncertainty values added to the mean changes are at 95% confidence intervals. Significant changes are marked in bold. Regions ED, SD, MD, LD and ND are shown in Fig. 1a. Region South combines ED, SD and MD, while region North combines LD and ND (shown in Fig. 3a). The Basin region is delineated in Fig. 1a with purple lines.

| **Annual Changes** | **2004-2005** | **2004-2006** | **2004-2007** | **2004-2008** |
|---|---|---|---|---|
| RH90 (m; ED) | -0.25±0.68 | **-0.88±0.69** | **-0.74±0.68** | **-0.86±0.68** |
| RH90 (m; SD) | -0.09±0.55 | **-0.59±0.53** | **-0.82±0.54** | **-1.08±0.54** |
| RH90 (m; MD) | -0.10±0.54 | -0.49±0.54 | **-0.92±0.54** | **-1.26±0.51** |
| RH90 (m; LD) | 0.09±0.77 | 0.08±0.77 | -0.05±0.76 | -0.49±0.79 |
| RH90(m; ND) | 0.09±0.91 | -0.14±0.92 | -0.07±0.94 | -0.30±0.93 |
| RH90(m, South) | -0.13±0.49 | **-0.60±0.48** | **-0.84±0.48** | **-1.11±0.48** |
| RH90(m, North) | 0.09±0.80 | -0.04±0.80 | -0.06±0.81 | -0.39±0.82 |
| RH90(m, Basin) | -0.07±0.44 | -0.45±0.45 | **-0.67±0.45** | **-0.82±0.45** |
| AGB (Mg/ha; ED) | -1.02±3.87 | **-4.71±3.70** | -3.80±3.83 | **-4.23±3.79** |
| AGB (Mg/ha; SD) | -0.15±2.93 | **-2.85±2.76** | **-4.05±2.73** | **-5.32±2.73** |
| AGB (Mg/ha; MD) | -0.59±3.05 | -2.55±2.92 | **-4.90±2.94** | **-6.76±2.94** |
| AGB (Mg/ha; LD) | 0.71±4.90 | 0.36±4.48 | 0.20±4.55 | -2.53±4.75 |
| AGB (Mg/ha; ND) | 0.62±5.42 | -1.05±4.73 | -0.16±5.23 | -0.76±4.92 |
| AGB (Mg/ha; South) | -0.49±2.66 | **-3.09±2.49** | **-4.34±2.46** | **-5.72±2.52** |
| AGB (Mg/ha; North) | 0.66±4.90 | -0.38±4.34 | 0.01±4.64 | -1.60±4.58 |
| AGB (Mg/ha; Basin) | -0.21±2.43 | -2.34±2.38 | **-3.41±2.40** | **-4.10±2.32** |
| Total Carbon (PgC; ED) | -0.03±0.11 | **-0.14±0.11** | -0.11±0.11 | **-0.12±0.11** |
| Total Carbon (PgC; SD) | -0.01±0.18 | **-0.18±0.17** | **-0.25±0.17** | **-0.33±0.17** |
| Total Carbon (PgC; MD) | -0.04±0.18 | -0.15±0.17 | **-0.29±0.18** | **-0.40±0.18** |
| Total Carbon (PgC; LD) | 0.06±0.42 | 0.03±0.38 | 0.02±0.39 | -0.22±0.40 |
| Total Carbon (PgC; ND) | 0.06±0.52 | -0.10±0.45 | -0.02±0.50 | -0.07±0.47 |
| Total Carbon (PgC; South) | -0.07±0.40 | **-0.46±0.37** | **-0.65±0.37** | **-0.86±0.38** |
| Total Carbon (PgC; North) | 0.12±0.88 | -0.07±0.78 | 0.00±0.83 | -0.29±0.83 |
| Total Carbon (PgC; Basin) | -0.05±0.63 | -0.60±0.62 | **-0.88±0.62** | **-1.06±0.60** |

**Supplementary Table 2**: Number of valid observations available in each screening step of GLAS data filtering over the Amazon forests.

| Screening steps | Points in 2003 | Points in 2004 | Points in 2005 | Points in 2006 | Points in 2007 | Points in 2008 |
|---|---|---|---|---|---|---|
| LC filter | 1313904 | 1488088 | 1579389 | 1397870 | 873773 | 772387 |
| VCF filter | 1023613 | 1177313 | 1243486 | 1105601 | 690725 | 602210 |
| Seasonal filter | 629965 | 558973 | 439072 | 377329 | 400586 | 225437 |
| Saturation filter | 597443 | 434098 | 369094 | 345785 | 382876 | 219133 |
| 2-peak filter | 149954 | 130795 | 83941 | 72436 | 76185 | 28312 |
| Cloud filter | 70759 | 65504 | 36876 | 34879 | 28564 | 8690 |
| Slope filter | 31134 | 26609 | 15679 | 16180 | 16578 | 5107 |

**Supplementary Table 3**. Summary of GLAS data filtering steps.

| Steps | Description |
|---|---|
| LC filter | Filter data using MODIS land cover map of 2005 and keep only waveforms located in pixels classified as EBF (evergreen broadleaf forests). |
| VCF filter | Further data screening using MODIS Vegetation Continuous Field (VCF) to retain the observations in dense forests only (VCF > 0.6). |
| Seasonal filter | Use GLAS observations in October and November to remove the potential seasonal effects |
| Saturation filter | Remove saturated waveforms using the Saturation Correction Flag (sat_corr_flg = 0) |
| 2-peak filter | Use waveforms with at least 2 peaks (local maxima) to represent reflectances from canopy and ground; Filter shots with zero amplitude for >1m in length between canopy and ground and ground peak <0.2V in waveform amplitude (indicating possibly false ground) |
| Cloud filter | Use cloud-free data only by checking the Atmosphere Characterization Flag (atm_char_flag = 0). |
| Slope filter | Keep only data with flat terrain (calculated slope less than 10 degrees) |

## Supplementary References

1. Harding, D. J. & Carabajal, C. C. ICESat waveform measurements of within-footprint topographic relief and vegetation vertical structure. *Geophys. Res. Lett.* **32,** 1–4 (2005).

2. Zwally, H. J., Schutz, R., Hancock, D. & Dimarzio, J. GLAS/ICEsat L2 Global Land Surface Altimetry Data (HDF5), Version 34. *Boulder Colo. USA NASA Natl. Snow Ice Data Cent. Distrib. Act. Arch. Cent.* (2014). doi:10.5067/ICESAT/GLAS/DATA211

3. Tropical Rainfall Measuring Mission (TRMM). TRMM (TMPA/3B43) Rainfall Estimate L3 1 month 0.25 degree x 0.25 degree, Version 7. *Goddard Earth Sciences Data and Information Services Center (GES DISC)* (2018). Available at: http://dx.doi.org/10.5067/TRMM/TMPA/MONTH/7. (Accessed: 16th June 2018)
